# Predicting the impact of promoter variability on regulatory outputs

**DOI:** 10.1038/srep18238

**Published:** 2015-12-17

**Authors:** Naomi N. Kreamer, Rob Phillips, Dianne K. Newman, James Q. Boedicker

**Affiliations:** 1 Division of Biology and Biological Engineering, California Institute of Technology, Pasadena, CA 91125, USA; 2 Department of Chemistry, California Institute of Technology, Pasadena, CA 91125, USA; 3 Division of Geological and Planetary Sciences, California Institute of Technology, Pasadena, CA 91125, USA; 4 Department of Applied Physics, California Institute of Technology, Pasadena, CA 91125, USA; 5 Department of Physics and Astronomy, University of Southern California, Los Angeles, CA 90089, USA; 6 Department of Biological Sciences, University of Southern California, Los Angeles, CA 90089, USA

## Abstract

The increased availability of whole genome sequences calls for quantitative models of global gene expression, yet predicting gene expression patterns directly from genome sequence remains a challenge. We examine the contributions of an individual regulator, the ferrous iron-responsive regulatory element, BqsR, on global patterns of gene expression in *Pseudomonas aeruginosa*. The position weight matrix (PWM) derived for BqsR uncovered hundreds of likely binding sites throughout the genome. Only a subset of these potential binding sites had a regulatory consequence, suggesting that BqsR/DNA interactions were not captured within the PWM or that the broader regulatory context at each promoter played a greater role in setting promoter outputs. The architecture of the BqsR operator was systematically varied to understand how binding site parameters influence expression. We found that BqsR operator affinity was predicted by the PWM well. At many promoters the surrounding regulatory context, including overlapping operators of BqsR or the presence of RhlR binding sites, were influential in setting promoter outputs. These results indicate more comprehensive models that include local regulatory contexts are needed to develop a predictive understanding of global regulatory outputs.

It is well appreciated that the rate of generation of new genome sequencing data is far outpacing our ability to make sense of it. For example, although considerable progress has been made in recent years to understand the roles of noncoding genomic regions^1^,^2^, our ability to apply this understanding in a predictive fashion is still quite limited. More fundamentally, it is not clear to what extent we can accurately predict genome-wide regulatory outputs from DNA nucleotide sequence, though many complementary approaches are being applied to decipher the regulatory logic in the genomes of both eukaryotes and bacteria^3^,^4^. Here, we put our understanding of how an individual bacterial transcription factor influences global gene expression to the test to explore the extent to which the binding sequence and the surrounding regulatory context tune promoter outputs.

Making precise predictions of genome-wide expression is hampered by an incomplete understanding of how regulatory information is encoded at different promoter regions^3^,^5^. We cannot accurately predict *a priori* what influence on gene expression a particular transcription factor will have at a given promoter. For the majority of transcription factors we only know where these factors are likely to bind and whether the transcription factor acts as an activator or repressor. Current models typically cannot quantitatively determine the magnitude of expression, nor predict how expression is modulated by the number of regulatory proteins per cell or the promoter architecture (defined as the sequence, orientation, location, and number of transcription factor binding sites, and their proximity to the RNA polymerase binding site). Even for well-characterized regulators, we are often surprised by experimental results contradicting expected trends^6^,^7^,^8^. A predictive understanding of how regulatory information is encoded in the genome would lead to more meaningful comparisons between genomes of related organisms, enhance our understanding of regulatory-genome changes associated with niche differentiation, and improve our ability to design synthetic regulatory networks.

We chose the ferrous iron [Fe(II)] responsive two-component system, BqsRS, from *Pseudomonas aeruginosa*^9^ to test our ability to quantitatively predict the regulatory output for an individual transcription factor. Most organisms require iron for essential cellular processes, including electron transfer steps in metabolism, but iron is a limiting nutrient in many environments^10^. Because iron uptake and localization are critical for growth and function, the cellular response to iron is complex and tightly regulated. In the body, our immune system controls pathogen proliferation in part by sequestering iron through high-affinity ferric iron [Fe(III)]-binding molecules such as lactoferrin^11^. In many environments, including the cystic fibrosis (CF) lung and soil, iron is present in both the Fe(II) and Fe(III) forms^12^,^13^. Furthermore, elevated Fe(II) in CF sputum correlates with severe disease states^12^. BqsRS consists of a sensor histidine kinase (BqsS) and a response regulator (BqsR) that responds specifically to Fe(II) at low micromolar concentrations in the periplasmic space^9^. BqsRS is known to be involved in rhamnolipid production and biofilm dispersal^14^ and it mediates cellular defenses against cationic stressors, including aminoglycoside and polymyxin antibiotics^15^. The consensus BqsR DNA binding motif^15^ is found in promoter regions of genes that are upregulated by BqsR, but it remains unclear whether BqsR binding alone is sufficient to elicit a regulatory response, or if the impact of BqsR depends on the context at each promoter.

One common model used to predict the influence of transcription factors on global gene expression is based on position weight matrices (PWM)^16^,^17^,^18^,^19^,^20^,^21^,^22^,^23^,^24^,^25^. Here, we used the previously derived PWM for the Fe(II) responsive transcription factor BqsR^15^ to search for potential binding sites throughout the genome. Hundreds of potential binding sites were found; RNA-Seq expression measurements revealed that the majority of genes containing potential BqsR operators were not strongly regulated by BqsR. This discrepancy suggests that either the PWM of BqsR does not accurately describe the interaction between BqsR and the genome, or that the surrounding operator context has a greater influence on promoter outputs than the local BqsR binding sequence. Through systematic measurements of how the architecture of BqsR containing promoters influences gene expression, we address how BqsR regulatory information is encoded in the genome and to what extent understanding the regulatory context is critical to develop a predictive understanding of the global effect of an individual transcription factor.

## Results

### Operator sequence diversity throughout the genome

**Figure 1:**
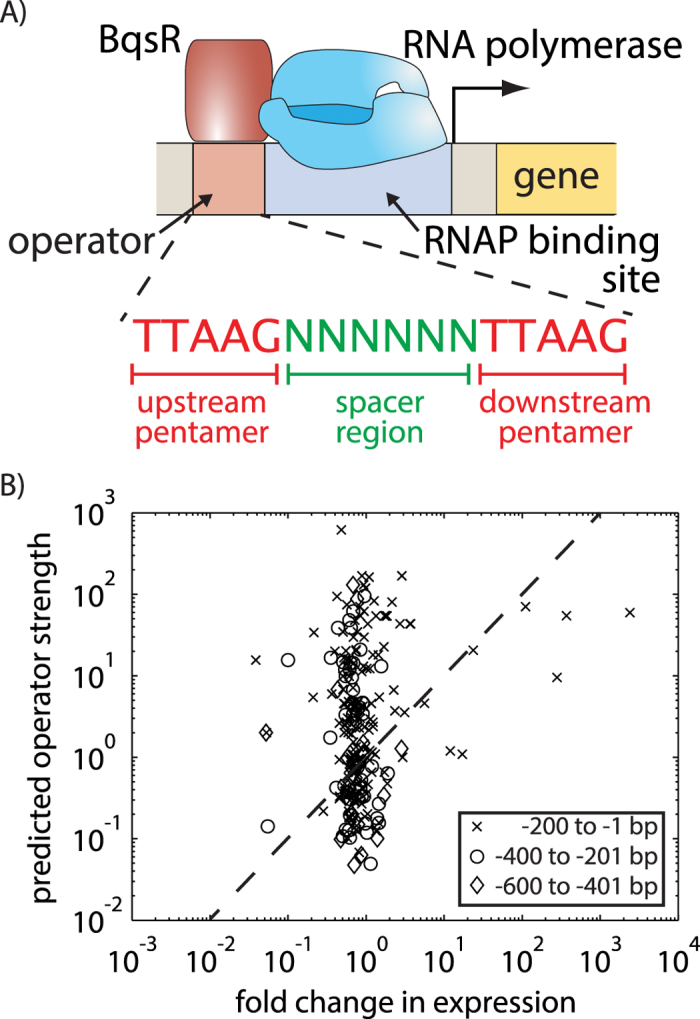
The BqsR binding motif in the genome and its impact on global expression. (**A**) The response regulator BqsR activates gene expression. The BqsR operator sequence contains a repeated pentamer (TTAAG) separated by 6 bp. (**B**) A comparison of the predicted operator strength with the observed experimental fold change in expression measured using RNA-seq. Operator strength was predicted with Equation 1 using best-fit values for parameters a and b. The curve shows y = x. For operator regions containing multiple BqsR binding sites, only the site with the highest score was plotted.

To construct a quantitative model capable of predicting the magnitude of gene expression directly for any given promoter region, we first dissected how variability in the BqsR operator modulates gene expression. BqsR is an activator that binds upstream of the gene transcription start site (Fig. 1A). Earlier work established the BqsR binding sequence contained a pair of highly conserved pentamers (Fig. 1A)^15^. There were 432 operators with 2 or fewer mutations in the pair of consensus repeated pentamers (TTAAG(N)_6_TTAAG), and over 5000 potential operators containing 3 mutations. This frequency of binding sites is not expected to occur by chance in the genome (Supplemental Fig. S1). Many potential BqsR operators were found within promoter regions throughout the genome, but it is not obvious at which of these operators BqsR binds and has a regulatory consequence.

The magnitude of BqsR-mediated gene expression for each of these potential operators can be predicted using a PWM for the operator binding site. A PWM uses a set of operator sequences to generate a DNA sequence motif reflecting the nucleotide frequency for each position in the sequence^16^,^17^. The PWM can then be used to rank order the regulatory strength of each potential operator. The operator strength is calculated using,


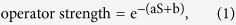


in which *S* is the operator score calculated using the PWM (see Position weight matrix calculations in Supplemental Material for further details), and *a* and *b* are parameters relating the PWM score to the operator affinity^17^. Operator strength is assumed to be proportional to the affinity of the operator for BqsR^26^,^27^,^28^.

Figure 1B compares the measured fold change in gene expression to the BqsR operator strength calculated using Equation 1, revealing a poor correlation between the PWM predictions and experimental expression. Scores were calculated for operators located in a promoter region, defined here as the region within 600 bp upstream of the protein coding sequence, using the PWM derived in^15^. The data in Fig. 1B was used to obtain values for parameters *a* and *b*, 1.6 and 1.9 respectively, which fall in the range of typical values^29^. These values appear in Equation 1 to relate the PWM score to the operator strength. The fold change in expression was calculated from RNA-seq data in WT and Δ*bqsR* strains grown anaerobically and shocked with 200 μm Fe(II), as reported in^15^.

Typically it is assumed that the rate of gene expression at a given promoter is proportional to the affinity for the transcription factor to the operator, known as the occupancy hypothesis^6^. The fact that several binding sites with high PWM scores were not induced by an Fe(II) shock raises several questions: how is regulator-binding specificity achieved; how does operator sequence modulate promoter outputs; and to what extent does the surrounding promoter context influence BqsR-mediated regulatory responses?

### Influence of the pentamer sequence on BqsR-mediated regulation

One potential cause of disagreement between model predictions and experimental measurements was that PWM did not accurately capture the relationship between operator sequence and binding affinity. To determine whether the PWM was missing key regulatory information, we experimentally dissected how the structure of the operator (*i.e.,* the sequence, length, orientation, and position) affected the level of gene expression. Although we analyzed operator diversity throughout the genome for clues as to which operator variations might impact BqsR-mediated expression, direct experimental measurements were used to construct our gene regulatory model. FIMO (Find Individual Motif Occurrences), part of the MEME Suite^30^, was used to identify potential BqsR binding sites in the genome. These potential operator sequences were further characterized by comparing the sequence, location, and orientation of the binding sites. The operator was split into three regions: the upstream and downstream pentamers and the spacer region (Fig. 1A). A small library of synthetic promoters was fused to the *lacZ* reporter gene and inserted into the genome at the *glmS* locus to quantify the influence of specific changes in promoter architecture on expression output. The synthetic promoter library was based on the BqsR binding sequence in the promoter for gene PA14_04180, the gene most highly upregulated by BqsR.

**Figure 2:**
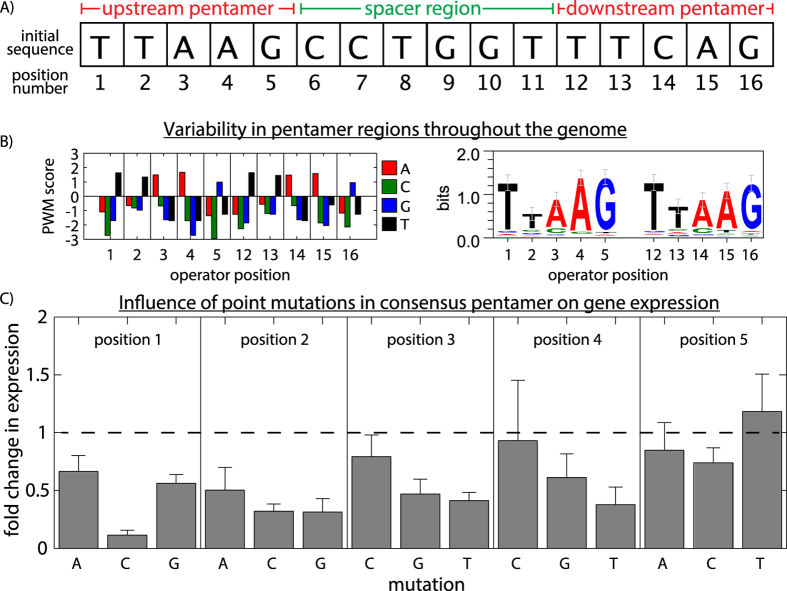
Variability of the BqsR operator’s repeated pentamer sequence and its influence on gene regulation. (**A**) The reference operator found in the PA14_04180 promoter region contains two pentamers separated by a 6-bp spacer region. (**B**) T he graph shows the PWM score and sequence logo for the upstream and downstream pentamer sequences calculated using the operators from Fig. 1. (**C**) Expression measurements of synthetic constructs quantified the influence of each point mutation for all nucleotides in the upstream pentamer on gene expression. Error bars show standard error of biological triplicates.

Previously, two repeated pentamers were found to be highly conserved and sufficient for BqsR binding^15^. The PWM score indicates the frequency of a given bp at each position in the sequence compared to the background distribution of bp in the genome [Supplemental Equations S1 and S2 and^16^,^17^]. Bases with a high score indicate a particular base being favored at a given position, and bases with negative scores indicate bases that are rare at a given position. The PWM of the pentamer regions was calculated using the 236 operator sequences from Fig. 1B containing up to 2 mutations in the pentamer regions (Fig. 2B). There is a non-uniform distribution of the scores, implying that some bases contribute more than others to the binding energy of BqsR to the operator.

A library of synthetic promoters based on a modified PA14_04180 promoter, shown in Fig. 2A, was constructed to test the influence of the pentamer sequence on gene expression. Only the upstream pentamer was mutated because the downstream pentamer overlaps the −35 region of the RNA polymerase (RNAP) binding site^15^. Additionally, the symmetry in the PWM scores in Fig. 2B suggests the two pentamer sequences may interact similarly with BqsR. The library contains all possible single point mutations for the upstream pentamer sequence, 15 constructs in total. In Fig. 2C, we report the gene expression level after Fe(II) shock for each mutant relative to the expression level of the wild-type promoter containing the binding site shown in Fig. 2A. Expression analysis of the promoter library revealed heterogeneity in the contribution of each position to the expression level. For example, the bases in position 5 have a weak influence on regulation, whereas mutating position 1 from T to C reduced expression nearly 10 fold. The large decrease in expression of this particular mutation may explain why a C in position 1 was rare in the potential binding sites found in the genome (Fig. 2B).

### Influence of the spacer sequence on BqsR-dependent regulation

**Figure 3:**
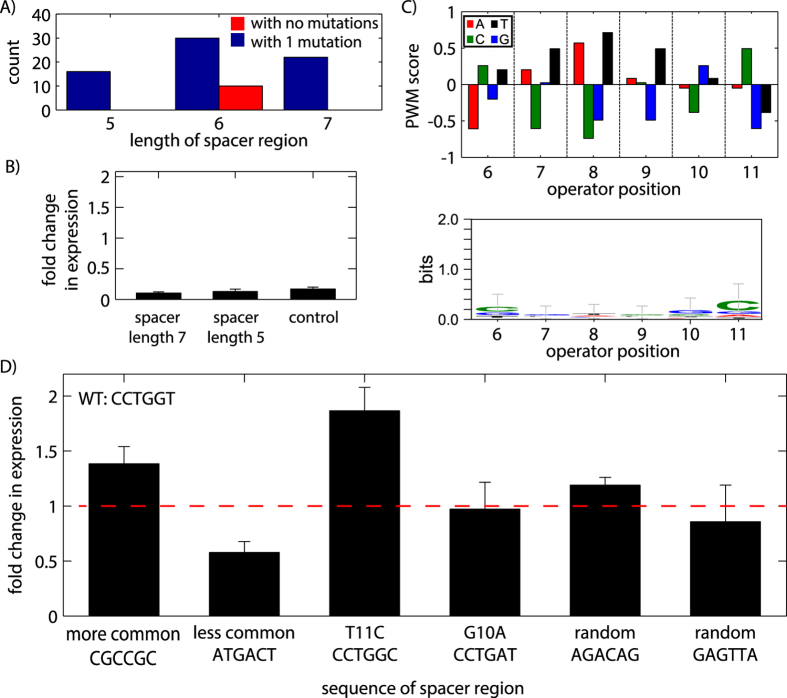
Variability of the spacer sequence and its influence on gene regulation. (**A**) The number of operators containing spacers of length 5, 6, or 7 bp is plotted for potential operators containing either 0 or 1 mutations in the pentamer regions, revealing a preference for spacer regions of length 6 bp. (**B**) Experimental measurements of synthetic operators confirm that a 6-bp spacer length is critical for BqsR-mediated regulation, as spacer lengths of 5 or 7 bp resulted in expression levels similar to the negative control in which the upstream pentamer was deleted. (**C**) Operators from (**A**) containing a 6-bp spacer region were analyzed for sequence preference by calculating the PWM score and sequence logo. (**D**) Gene expression measurements of 6 synthetic spacer sequences showed that the sequence of the spacer region modulates the level of gene regulation up to a factor of 2.

We also determined the natural variability of the spacer region between the two pentamers and its influence gene regulation. First, we examined the variability of spacer region lengths throughout the genome. All previously reported operator sequences had a spacer length of 6 bp, but it was unclear if spacer length modulated BqsR binding. Analyzing all the potential operators in the genome with spacer lengths of 5, 6 or 7 pairs shows that all the pentamer pairs with no mutations had 6-bp spacer regions, and that a spacer length of 6-bp was most common for operators with a single pentamer mutation (Fig. 3A). Indeed, spacer length is a key operator parameter. Spacers of length 5 or 7 lowered gene expression to less than 10%, an expression level similar to an operator for which the upstream pentamer has been deleted (control in Fig. 3B). Together these results suggest that a 6-bp spacer region is essential for operator binding. We also examined the influence of operator orientation on expression. The reverse or reverse complement of the upstream pentamer reduced expression to background levels (Supplemental Fig. S2).

The spacer PWM scores show that some bases occur frequently at specific positions, such as a C at position 11, although the sequence logo reveals a low information content of the spacer region (Fig. 3C). Synthetic promoters were created to quantify the influence of the spacer region on gene regulation. Because the number of potential spacer sequences was large (4^6^ = 4096), targeted changes were made to the spacer region based on analysis in Fig. 3C. Figure 3D shows measurements of gene expression from these constructs, revealing the spacer region sequence has the potential to moderately influence expression levels. For the six sequences measured, gene expression levels were found to vary up to a factor of 2.

### Binding site clusters

**Figure 4:**
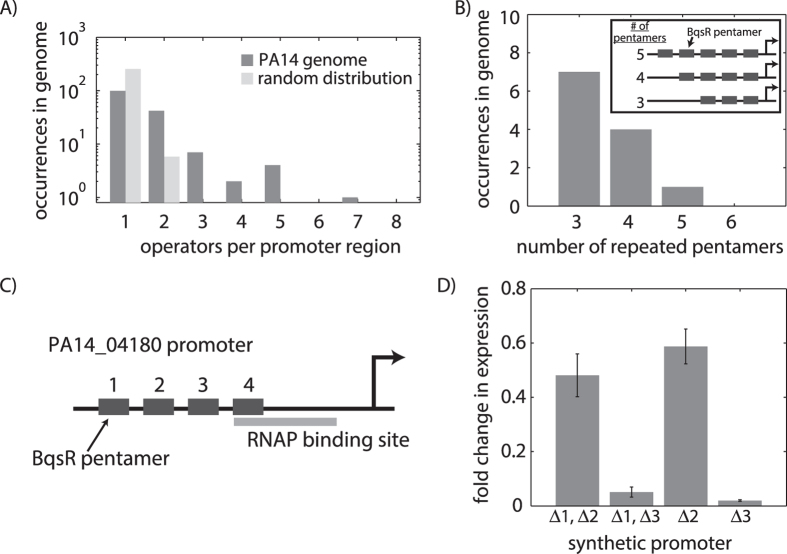
Promoter regions with multiple operators. (**A**) The number of operators within each promoter region of the genome as compared to randomly distributing the same number of operators throughout the genome. Operators are sequences 16 bp in length and contain up to 2 mutations in the pentamer regions. (**B**) Several of these clustered promoters are spaced by 6 bp, the same spacing between the two pentamer regions of an individual promoter, creating arrays of overlapping binding sites as shown in the schematic. (**C**) The promoter for PA14_04180 is one such promoter containing 4 repeated pentamer regions. (**D**) Gene expression measurements on synthetic versions of the PA14_04180 promoter, in which individual or pairs of pentamer repeats were removed, revealed that each pentamer repeat contributes to the overall level of gene regulation, although not all the BqsR binding sites contribute equally to the regulatory output.

Yet another aspect of promoter architecture is the number of binding sites in the promoter region. We searched the genome for operator clusters with a 6-bp spacer region between pentamers and only considered operators with a maximum of 2 pentamer mutations. The genome contained many promoters with multiple BqsR operators, up to a maximum of 7 potential operators in the same promoter region, which cannot be accounted for by a random distribution of binding sites (Fig. 4A). Some promoters contained overlapping operators that share a common pentamer sequence, creating multiple repeats of the pentamer sequence separated by a 6-bp spacer region with up to 5 repeated pentamers, as shown in the schematic of Fig. 4B.

We next experimentally dissected the role of operator clusters in the regulatory output. The native promoter region for gene PA14_04180 contains 4 proximal pentamers as shown in Fig. 4C (the truncated PA14_04180 promoter used in Fig. 2 contained only the 2 downstream pentamers). The most downstream pentamer, labeled 4 in Fig. 4C, is unique given that it overlaps the −35, RNAP binding site in the promoter. We created several synthetic promoters that lacked one or more pentamers, all of which retained pentamer 4 given its dual role in both BqsR and RNAP binding (Fig. 4D). A pentamer was deleted by mutating the pentamer sequence from TTAAG to ACTCA. As shown in Fig. 4D, BqsR binding to pentamers 3 and 4 was critical for strong expression. The supplemental binding sites 1 and 2 led to an additive increase in promoter output, but were not essential for expression.

### Deriving a binding energy matrix for BqsR

From the experimental dissection of BqsR-mediated gene expression, we constructed a BqsR activity matrix with a 6-bp spacer region to predict gene regulation for the entire genome. The activity matrix translates the operator nucleotide sequence to output gene expression. Because gene expression is assumed to be proportional to operator affinity, this matrix is referred to as a “binding energy matrix”. Such binding energy matrices have been developed previously for other transcription factors^22^,^31^, and in the case of the Lac repressor in *E. coli* have been shown to accurately predict a wide range of promoter outputs^32^. We assumed an additive contribution of operator clusters, operator occupancy was linearly proportional to the change in expression level, and stronger BqsR binding increased transcription.

For the pentamer region, measurements of the fold change in expression from Fig. 2C were converted to a change in binding energy using,


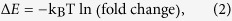


where k_B_ is Boltzmann’s constant, T is the temperature, and ΔE is the change in the binding energy of BqsR to the operator (see Supplemental section “Predicting gene expression from operator sequence” and Figs S3 and S4 for further details). For the spacer region, a “best fit” binding energy matrix was fit to the expression data for the 7 variants of the spacer sequence measured in Fig. 3D (see Supplemental Materials section “Deriving the energy matrix for the spacer region” and Fig. S5).

**Figure 5:**
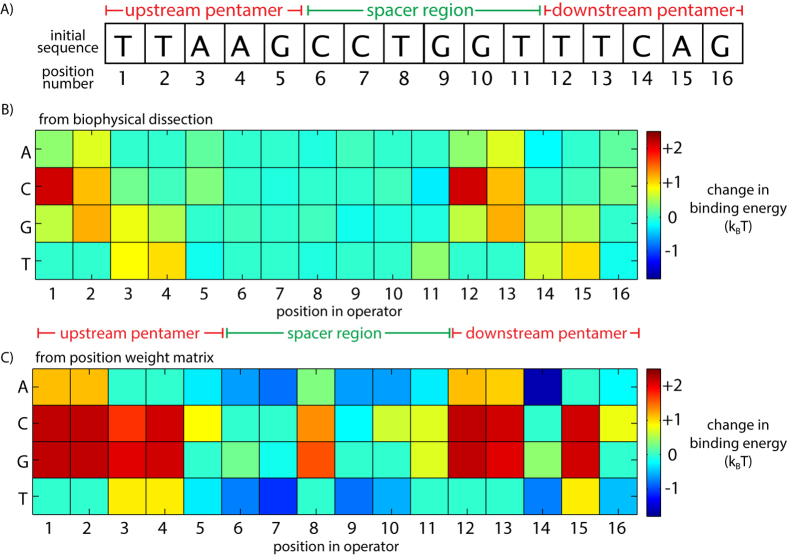
Creating a binding energy matrix for the BqsR operator sequence. (**A**) The reference operator sequence from gene PA14_04180. (**B**) Energy matrix derived from the experimental measurements of regulation by synthetic operators. (**C**) For comparison, the energy matrix derived from the position weight matrix used for prediction shown in Fig. 1B, which was constructed using qPCR expression data for 12 genes. Binding energies in k_B_T units are relative to the binding strength to the reference operator in (**A**). Matrix positions in dark red indicate bp changes that cause large reductions in expression levels, whereas matrix positions in dark blue indicate bp that increase expression levels.

The final 16-mer energy matrix reported the change in binding energy for any operator sequence relative to the initial sequence (Fig. 5B). Using this matrix we predicted the expression of a given promoter relative to a promoter containing the reference operator (Fig. 5A). For comparison, Fig. 5C showed the binding energy matrix derived from the PWM, scaled using the parameters *a* and *b* as in Fig. 1B. The PWM method of constructing an energy matrix differed from ours, in that we did not assume that the consensus sequence in the genome has the strongest binding energy. Although we analyzed potential operators throughout the genome for clues as to what operator parameters might influence regulation, ultimately, our matrix was based on direct, quantitative experimental measurements of gene expression from a library of synthetic operators containing *lacZ*-promoter fusions.

### Global prediction of BqsR-mediated gene regulation

With these operator rules and the experimentally derived energy matrix (Fig. 5B), we made new predictions for BqsR-mediated gene regulation to test if the PWM missed key regulatory information encoded in the primary DNA sequence. If sequence information was missing from the PWM approach, predictions derived from Fig. 5B will significantly improve our predictive capability. The fold change in gene expression due to each potential operator in the genome was calculated using Equation 2 (see Supplemental Figs S6 and S7 for the distribution of predicted operator affinities). For promoter regions containing multiple BqsR binding sites, we assumed each operator acted independently and the total fold change was the sum of the fold change for each individual operator (see Supplemental section “Additive approximation for multi-operator promoters” for more details). Since it is known that the position of the operator relative to the transcription start site plays a role in regulatory outputs, we only included potential operators up to 600 bp upstream from the protein coding sequence. Additionally, genes were predicted to be BqsR-regulated if they contain a maximum of 3 mutations in the 10 base pairs encoding the upstream and downstream pentamers, given that most mutations only moderately influenced expression (Fig. 2C).

**Figure 6:**
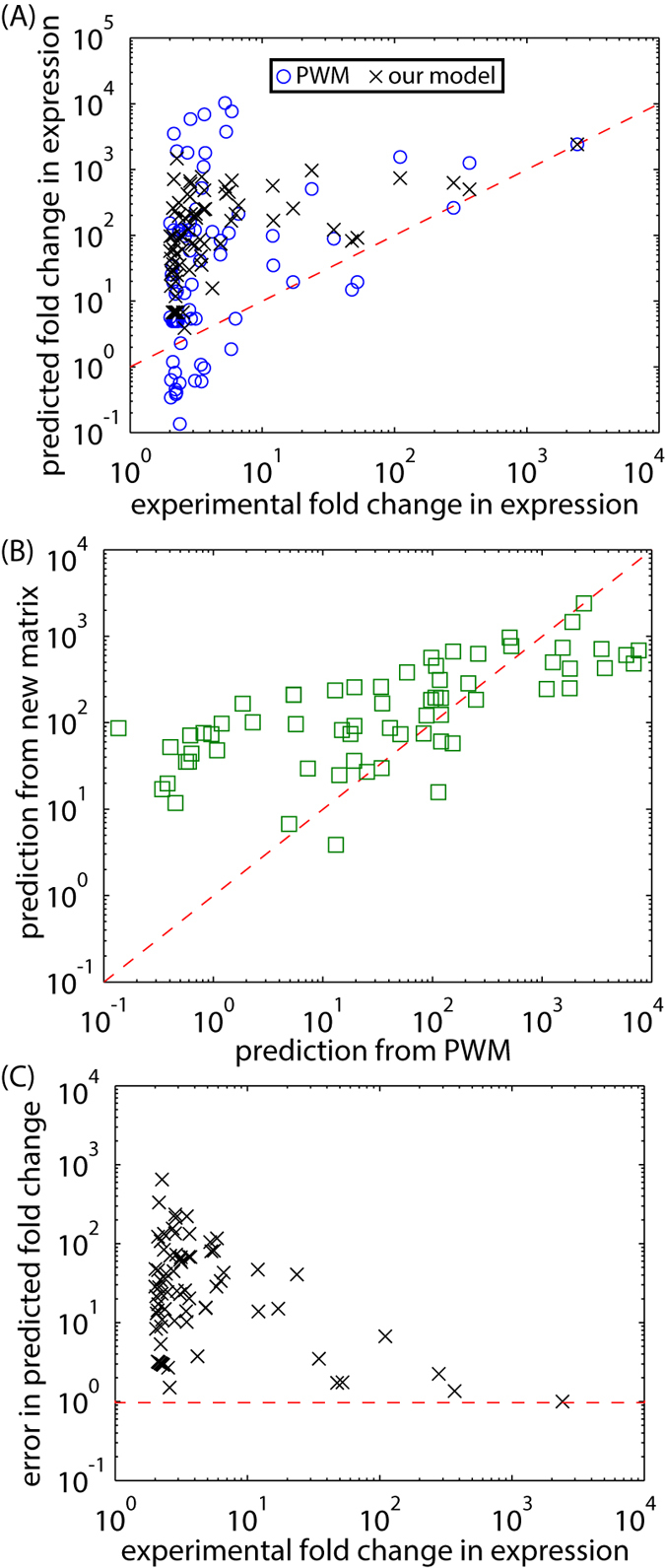
Comparison of global predictions of gene expression using the energy matrices in Fig. 5 to gene expression measured using RNA-Seq. (**A**) Predicted vs. experimentally measured BqsR-mediated fold change in expression levels for 75 genes. Black x’s show predicted expression based on the energy matrix shown in Fig. 5B. Blue circles show predicted expression based on the PWM shown in Fig. 5C. (**B**) Both PWM model and our model made similar predictions of fold change in expression. (**C**) Error in prediction, defined as the ratio of predicted to experimentally measured fold change in gene expression, plotted as a function of measured fold change in expression.

These predictions were compared to RNA-Seq measurements of global BqsR-mediated gene expression reported previously^15^. As a conservative annotation of BqsR-regulated genes we compare transcriptional units [regions which may contain several genes that are co-transcribed as defined by Wurtzel *et al.*^33^ whose expression was changed 2-fold or greater in WT compared to the ∆*bqsR* mutant in response to Fe(II) shock. Figure 6A shows comparisons of the predicted fold change in expression for 75 transcriptional units, predicted using either our binding energy matrix (Fig. 5B) or the PWM derived binding energy matrix (Fig. 5C), to the experimentally measured fold change in expression. All predictions were normalized to the expression level of the reference gene, PA14_04180. The predictions from our model and the model based on the PWM were similar with our model predicting stronger expression for weak potential binding sites (Fig. 6B). Additionally, our model predictions were accurate for promoters giving a strong response, greater than 25 fold, to ferrous iron shock in experiments (Fig. 6C). These results suggested the energy matrix accurately predicted operator occupancy. Our inability to predict the regulatory influence of BqsR at most promoters was not rooted in a misunderstanding of BqsR binding, instead, for most genes the surrounding regulatory context of each promoter was more important than operator affinity in setting expression levels.

### A closer look at genes that were poorly predicted

Both energy matrices in Fig. 5 poorly predicted BqsR-mediated regulation for the majority of the transcriptional units containing predicted BqsR operators. To explore why this might be, we examined the broader regulatory context of each promoter to determine whether the model overlooked key inputs into BqsR-mediated regulatory decisions. One parameter ignored in our initial predictions was the position of the BqsR operator relative to the gene transcription start site (TSS). The results shown in Fig. 6 consider operator position relative to the protein coding sequence (CDS), only including BqsR binding sites within 600 bp upstream of the protein coding sequence. However, the position of the BqsR binding site relative to TSS as opposed to the CDS is more relevant to transcriptional regulation^6^.

**Figure 7:**
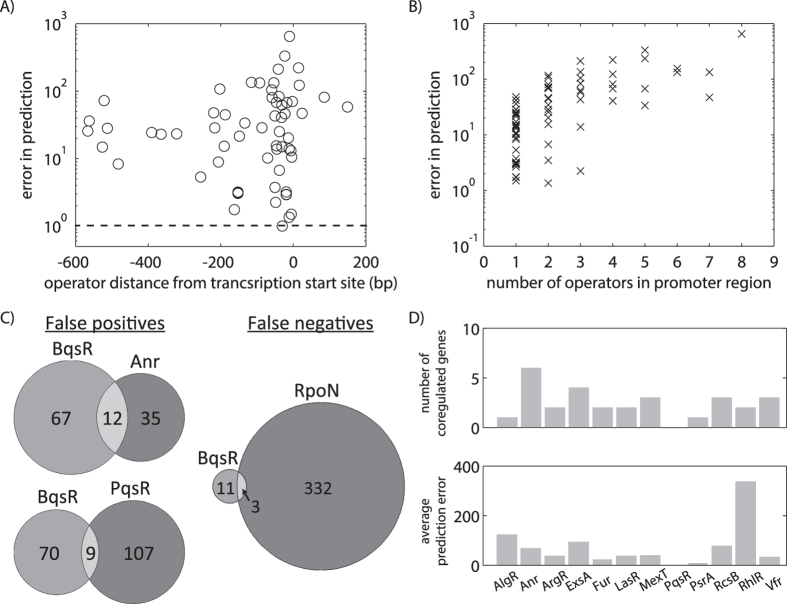
The influence of operator position and secondary transcription factors on the ability to predict BqsR-mediated gene regulation. (**A**) For the genes predicted in Fig. 6, the error in prediction, defined as the ratio of predicted over experimentally measured expression levels, is shown as a function of operator positions relative to the transcription start site. A prediction error of 1 indicates the measurement exactly matched the prediction. (**B**) Error in prediction plotted as a function of the number of BqsR binding sites found in the promoter region. (**C**) Venn diagrams showing regulon overlaps between the predicted genes and selected transcriptional regulators with statistically significant overlap. See section “Comparison to other operons” in Supplemental Materials. (**D**) Position weight matrices for 12 transcription factors were used to identify which of the predicted genes were likely to be coregulated by an additional transcription factor. The upper graph shows the number of promoter regions containing a potential binding site for a second transcription factor. Bars on the bottom graph show the average prediction error for each set of genes containing a secondary transcription factor; the larger the ratio, the greater the error.

To gauge the influence of the spatial relationship between the TSS and the BqsR binding site on gene expression, the distance between the TSS and the BqsR operators was examined. Figure 7A showed the ratio of the predicted expression level to the experimentally measured expression level as a function of operator position relative to the TSS. The TSS for 62 out of the 75 transcriptional units predicted in Fig. 6 could be determined from RNA-Seq data or were known^33^. A ratio near 1 indicates an accurate prediction, with higher values signifying greater error. Although it was interesting that most of the potential operators were found near the TSS, Fig. 7A indicated predictability was not correlated with operator position. The transcriptional units most accurately predicted had operators located within 200 bp upstream of the TSS, as would be expected for a typical activator in bacteria^34^.

In general we predicted a higher level of expression than experimentally observed, potentially caused by our assumption of additive contributions from multiple operators in the same promoter region. To analyze the impact of operator number on predictive ability, we examined the ratio of predicted to measured expression as a function of the number of putative operators in the promoter region (Fig. 7B). Each individual BqsR operator contained two pentamer regions separated by 6 bp with any number of nucleotides allowed between the 16-mer operators. The results reveal a trend of poor predictions for promoter regions containing multiple BqsR binding sites. For promoter regions containing up to three operators, predictive ability varied, but in general predictions were within 50 fold of measured values. For promoter regions containing 4 or more binding sites, predictions were typically higher than experimental measurements by a factor of 50 or more, with the poorest predictions occurring for the promoter containing 8 operators. These results suggest that the assumption that operators behave independently may not be valid for promoter regions containing many operators, and that some operators in large clusters of binding sites may be nonfunctional. Further analysis showed the additive model used above had similar predictions to a thermodynamic model derived for a promoter containing two operators (Supplemental Figs S8 and S9). Predictive ability also did not improve when considering only the strongest or most downstream operator at each promoter (Supplemental Fig. S10), or when taking operator orientation relative to the direction of gene coding sequence into account (Supplemental Fig. S11).

### Role of additional transcription factors in regulation

We next examined whether the poorly predicted transcriptional units had other known transcription factor binding sites in their promoter regions. To address this, we took a two-pronged approach. We used the PWMs reported for the 12 *P. aeruginosa* transcription factors annotated on the Prodoric database^24^. However, due to our findings that PWMs vastly overpredict potential operators, we also compared our predicted regulon (defined as the genes under BqsR control) with 13 experimentally validated *P. aeruginosa* regulons^35^,^36^,^37^,^38^,^39^,^40^,^41^,^42^,^43^,^44^,^45^,^46^,^47^,^48^.

Only two transcription factors, Anr^35^ and PqsR^42^,^43^, had experimentally determined regulons that had a statistically significant overlap with the transcriptional units predicted to be upregulated by BqsR but which were not upregulated in the RNA-Seq data (Fig. 7C “false positives” and Supplemental Table S1). PqsR controls the *Pseudomonas* quorum sensing regulon that responds to PQS; Dong *et al.*^14^ showed the concentration of PQS is reduced in the ∆*bqsR* mutant. Cells were grown in anaerobic conditions to ensure Fe(II) remained stable, the condition where Anr is active. Figure 7C “false negatives” also shows overlap of the RpoN^44^ regulon with transcriptional units whose expression levels changed as a result of the iron shock, but were not predicted. RpoN encodes an alternate sigma factor induced in stationary phase^49^ and under nitrogen limitation, and has been shown to influence quorum sensing regulation^50^. The cells used in the RNA-Seq experiment were harvested in late stationary phase. All of the overlapping genes predicted showed upregulation by RpoN.

The PWMs from the Prodoric database were used to predict potential transcription factor binding sites throughout the *P. aeruginosa* PA14 genome. In an attempt to limit the error associated with PWM-based predictions, for each transcription factor we assumed only the most probable 100 binding sites were capable of having a regulatory influence. Overall we identified potential secondary regulators in 25 of the 75 predicted transcriptional units (Fig. 7D). To gauge whether the presence of specific transcription factors led to poor predictions of gene expression, the average prediction error, the predicted fold change in expression divided by the experimental fold change in expression, was calculated for all the genes containing a secondary transcription factor (Fig. 7D). Genes containing the transcription factors Fur, PsrA, and Vfr were all predicted well, despite the potential influence of these additional regulators. Expression measurements for genes containing AlgR, Anr, ExsA, RcsB, and RhlR in their promoter regions deviated most from predictions. Because the presence of a potential RhlR binding site in promoter regions caused the most deviation between predictions and experimental measurements, the effect of RhlR on BqsR-mediated gene expression was examined in further detail.

### Measuring gene expression from promoters coregulated by both BqsR and RhlR

**Figure 8:**
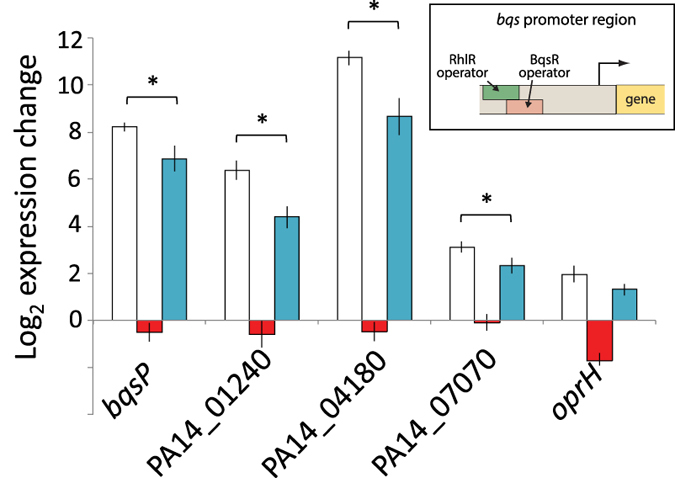
Fold change in expression due to ferrous iron shock for promoters coregulated by both BqsR and RhlR. For genes containing both BqsR and RhlR binding (bqsP, PA14_01240, PA14_04180, and PA14_07000), the fold change in expression due to Fe(II) shock was decreased upon overexpression of RhlR, demonstrating the ability of RhlR to modulate the effect BqsR has on expression at these promoters. Expression from the *oprH* promoter, which does not contain an RhlR binding site, was not significantly influenced by RhlR overexpression. The inset shows the *bqs* promoter, which contains overlapping operators. WT response is shown in white, *∆bqsR* is shown in red, and WT overexpressing *rhlR* is shown in blue. *indicates a p-value < 0.05.

Prodoric was used to search promoter regions of BqsR-regulated genes for potential RhlR binding sites. Intriguingly, the RhlR binding motif overlapped with the BqsR binding site in many of the promoters. In the *bqs* promoter the RhlR binding site overlaps with the upstream BqsR pentamer and the downstream BqsR pentamer overlaps with the −35 RNAP binding site. In these overlapping promoters, because RhlR and BqsR cannot bind simultaneously, high levels of RhlR should competitively exclude BqsR and thus lower expression. Ordinarily the quorum sensing gene *rhlR* is upregulated in response to an autoinducer indicative of high cell density during stationary phase. However, in the absence of autoinducer, RhlR can act as a repressor^51^. An *rhlR* overexpressing strain was used to express RhlR in early exponential phase. To determine the effect of RhlR on *bqsR* expression, qPCR analysis was used. Figure 8 shows BqsR-dependent expression of genes predicted to have an RhlR binding site. For all genes, the RhlR overexpressing strain showed a statistically significant (p-value ≤ 0.05 by unpaired two-tailed t-test) decrease in expression compared to wildtype. For *oprH,* a gene containing a BqsR-responsive operator but not a putative RhlR binding site, expression did not significantly change when RhlR was overexpressed. RhlR significantly changes the regulatory influence of BqsR in promoter regions containing BqsR and RhlR operators.

## Discussion

Using the *P. aeruginosa* Fe(II) responsive regulator, BqsR, as a test case, we examined our ability to make quantitative predictions about the influence of an individual transcription factor on global gene expression levels. A PWM model did not accurately predict global gene expression patterns, leading us to hypothesize that either the PWM model did not capture how operator affinity was encoded in the operator sequence, or that the regulatory influence of BqsR was dictated by the surrounding regulatory context of each promoter. A detailed model of operator binding through a synthetic promoter library revealed that predicting transcription factor affinity alone was insufficient to predict the global expression levels. Although clusters of overlapping operators had a combined impact on regulatory outputs, promoters containing large numbers of potential operators were poorly predicted by the model. The proximity to the transcription start site also did not correlate with predictive ability, despite the most upregulated genes containing potential operators within 200 bp of the transcription start site. These finding suggest that secondary regulators were important in determining the influence of BqsR on expression levels at promoters throughout the genome, as supported by the impact of RhlR in modulating BqsR-mediated expression.

While our model of operator occupancy outperformed the original PWM-derived model, the improvement was modest. Similarity between our binding energy matrix and the PWM supports the ability of PWM to describe operator affinity, at least when enough operator sequences can be identified to calculate an accurate binding matrix. However, our results underscore that caution should be used in relating operator strength to expression levels. Many genes highly upregulated in experiments were accurately predicted (within a factor of 3), but many operators with binding strengths predicted to be similar to the most upregulated gene (PA14_04180) had weak or immeasurable expression. This type of disagreement between experimental measurements and predictions highlights how difficult it is to make reliable predictions of gene expression directly from the genome sequence, and call attention to the need to more systematically study the influence of promoter diversity on expression.

That the broader regulatory context at each promoter may control the influence of individual transcription factors is not a new idea. Several studies have attempted to predictively integrate inputs from multiple transcription factors at a single promoter, however none of these studies used their findings to predict expression for additional promoter contexts within the genome^52^,^53^,^54^,^55^. DNA structure is another aspect of the regulatory context that modulates promoter outputs. Genome shape and mechanics, such as nucleosome wrapping and DNA loop formation, mediate both transcription factor binding and interactions^3^,^8^,^56^. DNA can also mediate allosteric effects between adjacent transcription factors^57^. Regulatory interactions have also been explored from a systems perspective^58^. One study reported that only 60% of the interactions between regulators could be accurately predicted in *E. coli*^4^. Despite careful studies on many aspects of the broader regulatory context in several systems, it remains unclear to what extent a general framework can predict the influences of promoter diversity on regulation.

Moving forward, we can leverage the lessons learned here by examining in more detail remaining questions. For example, several promoters have large clusters of binding sites, unlikely to be present by pure chance, however our predictive ability decreased with increasing cluster size. Perhaps under the conditions measured, weaker operators have a low probability of occupancy and therefore do not contribute to regulation^59^, although how weak an operator must be before it no longer modulates expression level is unclear, and may be context dependent^60^. By examining a broader set of expression conditions, we may be able to develop a set of rules that predict when and how the number of operators in the clusters is significant. Additionally, we should transition from the bioinformatic analysis presented here to rigorous experimental quantification of the role multiple transcription factors play in modulating promoter outputs. Future quantitative regulatory models should incorporate feedback in the dynamics of transcription factor levels. Bacteria respond to a wide variety of external stimuli, offering useful model systems in which to understand the logic and mechanisms of signal integration at the promoter level. Such work would complement ongoing efforts in synthetic and developmental biology^55^,^58^,^61^.

## Methods

### Growth media and culturing conditions

*P. aeruginosa* PA14 was grown both aerobically and anaerobically at 37 °C in MOPS minimal medium (MOMM) in acid washed glassware to ensure cells were Fe-limited. The basic MOMM is composed of 40 mM C_4_H_4_Na_2_O_4_ · 6 H_2_O, 9.3 mM NH_4_Cl, 2.2 mM KH_2_PO_4_, 25 mM KNO_3_, 25 mM NaNO_3_, 25 mM MOPS, 25 mM NaMOPS pH 7.2. Additionally, immediately prior to inoculation 100 μM CaCl_2_, 1 μM (NH_4_)_2_Fe(SO_4_)_2_ 6 H_2_O, 1 mM MgSO_4_, and trace metals were added^62^. Any composition changes are noted. All PA14 cultures were prepared by inoculation of MOMM media with the desired strains for 16 hours overnight shaking aerobically then grown anaerobically in a coy chamber with an atmosphere of 80% N_2_, 15% CO_2_, and 5% H_2_ at 37 °C.

### Strain construction

The strains used in this work were constructed from the wild type strain *P. aeruginosa* UCBPP-PA14. To monitor gene expression in these strains, the gene construct containing the versions of the PA14_04180 promoter attached to the *lacZ* reporter gene were inserted into the genome. Briefly, Gibson assembly was used to insert a gene construct containing 530 bases of the wildtype PA14_04180 attached to *lacZ* between the transposon sites of the plasmid pUC18T-mini-Tn7t. The region between the transposon sites contained a selection marker for growth on gentamicin. This base construct was then mutated using site directed mutagenesis to create synthetic versions of the promoter. Gene constructs were transferred into the *glmS* locus of the genome of *P. aeruginosa* using triparental mating^63^. Once inserted, constructs were verified using Sanger sequencing.

### Measuring gene expression of mutant library in response to iron shock

The gene reporter *lacZ* quantified the change in gene expression in response to changes in ferrous iron. Anaerobic cultures grown to final OD_500_ of 0.2–0.3 were uncapped inside of an anaerobic chamber and aliquoted to 1.7 ml tubes containing a final concentration of 400 μM ferrous iron (solutions of FeNH_4_SO_4_). Shocking with 400 μM Fe(II) more rapidly induced the maximal BqsR-mediated regulatory response observed with 200 μM Fe(II), which was used in previous RNA-Seq experiments^9^ (Supplemental Fig. S12). Fe(II) has been measured approaching 300 μM in CF sputum^12^. A ferrozine assay was used to confirm the concentration of the stock ferrous iron solution. The ferrous iron treatment was performed in triplicate for each strain measured. Cells were incubated in the anaerobic chamber at room temperature for 4 hours and then transferred to a 96 well plate for measurement of gene expression. Each well received 150 μl of ferrous iron treated cells with 50 μL of a media containing 50 ng/ml of the fluorogenic LacZ indicator fluorescein di-β-D-Galactopyranoside (FDG, Marker Gene Technologies, Inc.). OD_550_ and fluorescence with an excitation and emission of 490 nm and 520 nm respectively were measured in each well under anaerobic conditions every 5 minutes for 1 hour with a BioTek Synergy 4 plate reader. See Supplemental Fig. S12 for control experiments regarding this procedure.

To calculate the change in gene expression after Fe(II) shock, the background corrected fluorescence measurements were divided by the background corrected absorbance measurement to quantify the gene expression per cell. To calculate the fold change in gene expression for a given strain, the gene expression measurement was divided by the expression from a strain containing the reference BqsR operator shown in Fig. 2A, the downstream operator of the PA14_04180 gene.

### RhlR and BqsR co-regulon prediction

The position weight matrix for the RhlR DNA binding site^40^ was input into FIMO^30^, a tool which searches for a consensus sequence within a database. In this case, the database supplied was the 500 bp upstream from the translation start site for genes in the BqsR regulon.

### Effect of RhlR on Fe(II) shock conditions

Aerobic cultures of *P. aeruginosa* WT-pMQ72^64^, ∆*bqsR*-pMQ72, and WT-pMQ72-*rhlR* were grown in 3 ml MOMM supplemented with 100 μg/ml gentamycin at 37 °C for 36 hours. Anaerobic cultures were grown in 20 ml MOMM supplemented with 100 μg/ml gentamycin and 1% arabinose (to induce *rhlR* expression) with 1% inoculum from aerobic overnight culture. When the cells reached early exponential phase (Beckman spectrophotometer 20; OD_500_ = 0.2), 9 ml of culture was removed. 4.5 ml culture was added to 9 ml of RNAprotect (Qiagen) before and after a 30 minute 200 μM ferrous ammonium sulfate shock at room temperature. The cells were incubated with RNAprotect for 5 minutes and centrifuged for 10 minutes at 5000 × g. The supernatant was discarded and the pellets stored at −80 °C.

### mRNA isolation and qPCR data analysis

mRNA was isolated from stored cell pellets using the RNeasy kit mini (Qiagen) with optional on-column DNA digestion according to the manufacturer’s instructions. Subsequently, the RNA was treated with TURBO DNA-free (Applied Biosystems). cDNA was generated with iScript (Bio-Rad) random-primed reverse transcriptase reaction following the manufacturer’s protocol. An mRNA genomic contamination control and cDNA was used as template for quantitative-reverse transcriptase-PCR (Real Time 7500 PCR Machine, Applied Biosystems) using iTaq Universal SYBR Green Supermix (Bio-Rad). Samples were assayed with 3–5 biological replicates. *recA* and *clpX* were used as endogenous controls^65^. Fold changes were calculated using the ΔΔC_t_ method^9^. To ensure *recA* was constant in all conditions tested, the relative fold change was measured for the internal control *clpX,* whose expression was also expected to remain constant across all our treatments. Only those samples with a *clpX* fold change between 0.5–2 were used. Log_2_ of the final fold change was reported. Results were compared with an unpaired 2-tailed t-test assuming unequal variances.

### Ferrozine assay

This colorimetric assay measures Fe(II) concentration. The Stookey method^66^ was modified for 96-well plate format. All measured Fe(II) concentrations were within 5% of reported value.

### Bioinformatics

To analyze potential BqsR binding sites in the genome, occurrences of the motif (TTAAG(N)6TTAAG) were found in the genome of Pseudomonas aeruginosa PA14 using the program FIMO (Find Individual Motif Occurrences), part of the MEME Suite30. Motif occurrences were then sorted and analyzed using Matlab. The same process was used to locate binding sites of other transcription factors using binding motifs listed in the Prodoric database24. Sequence logos were calculated using WebLogo 3.4.

### Comparison to the other operons

From published microarray and RNA-Seq papers^35^,^36^,^37^,^38^,^39^,^40^,^41^,^42^,^43^,^44^,^45^,^46^,^47^,^48^ regulons for other transcription factors were defined. The list of genes in the transcription factor regulons were converted to transcriptional units^33^. Comparisons between transcriptional units (TU) in the regulon to two lists were made to discover the number of shared TUs: TUs in prediction but not observed in RNA-Seq data and TUs in RNA-Seq data but not predicted. In R, a hypergeometric test assigned a p-value to the overlapping regulons. For those regulons with significant overlap, whether the TUs were upregulated or downregulated was noted. See Supplemental Material for further details.

## Additional Information

**How to cite this article**: Kreamer, N. *et al.* Predicting the impact of promoter variability on regulatory outputs. *Sci. Rep.* **5**, 18238; doi: 10.1038/srep18238 (2015).

## Supplementary Material

Supplementary Information
