# Supplementary material for: Predicting the impact of promoter variability on regulatory outputs
Source: Sci Rep. 2015 Dec 17;5:18238. doi: 10.1038/srep18238 (PMC4682146; doi:10.1038/srep18238)
Supplement: Supplementary Information [file srep18238-s1.pdf]

Predicting the impact of promoter variability on regulatory outputs

Naomi Kreamer, Rob Phillips, Dianne Newman and James Boedicker

Supplemental Materials

Table of Contents

Position weight matrix calculations	2
Bioinformatic analysis of the genome	2
Estimates of number of potential binding sites	2
Influence of pentamer orientation on BqsR-mediated gene regulation	3
Distribution of Binding Sites in the Genome	4
Predicting gene expression from operator sequence	4
Deriving the energy matrix for the spacer region	8
Distribution of operator strengths and gene expression	9
Additive approximation for multi-operator promoters	9
Single operator predictions	12
Gene expression predictions taking into account operator orientation	12
Measuring gene expression in response to ferrous iron shock	13
Comparison to the other operons	13
Supplemental Material References	14

Position weight matrix calculations

Position weight matrix (PWM) calculations are performed following [1, 2]. From a list of N potential operator sequences, the frequency of each basepairs at each position is calculated using,

$$p_{xi} = \frac{n_{xi}+1}{N+4}, \qquad \text{(Equation S1)}$$

where p_{xi} is the estimated probability of having nucleotide x at position i, n_{xi} is the number of sequences containing nucleotide x at position i. The +1 in the numerator ensures that $p_{xi} > 0$.

From these frequencies and given the background frequency of GC in the genome as 66% [3], the score (S) of any potential operator sequence of length L is calculated using,

$$S = \sum_{i=1}^{L} ln \frac{p_{xi}}{q_x}, \qquad \text{(Equation S2)}$$

where q_x is the background frequency of the nucleotide in position x. The score can be translated to the binding energy of BqsR to a given operator sequence using Equation 1, with greater scores indicating stronger binding. Score as defined in Equation S2 is used in Figures 1B, 2B, and 3C.

To determine how a change in the operator sequence influences the BqsR binding energy, the score of the mutated operator is subtracted from the score of the original operator.

It should be noted that throughout the Supplemental Materials we analyze operator sequences in the genome found using the position weight matrix. These sequences are potential or predicted operators, given that they have not been experimentally confirmed. The only operators experimentally confirmed in this work are those found in the promoter region of gene PA14_04180.

Bioinformatic analysis of the genome

FIMO (Find Individual Motif Occurrences), part of the MEME Suite [4], was used to find potential BqsR binding sites in the genome of *Pseudomonas aeruginosa* strain UCBPP-PA14. An input sequence of TTAAG(N6)TTAAG was used with a threshold p-value of 0.1, resulting in over 80,000 output sequences. A large p-value threshold was used to include all potential operator sequences.

Custom Matlab code was used to sort through the FIMO output sequences. Each sequence was mapped onto the genome to determine the orientation and position of each sequence relative to the coding sequences of each gene. Sequences not falling within 0 to -600 basepairs of a coding sequence were discarded. Mutations in the pentamer regions of each sequences were then identified, as compared to TTAAG(N6)TTAAG. To find sequences with spacer lengths of 5 or 7, the input sequence to FIMO was adjusted to TTAAG(N5)TTAAG or TTAAG(N7)TTAAG respectively.

Estimates of number of potential binding sites

The number of binding sites expected within a genome of size 6,537,647 basepairs was calculated. Each binding sites has a specified number of mutations within the 10 basepairs of the repeated pentamer region and is separated by 6 random basepairs. These random binding sites can occur anywhere within the genome and have either orientation. For example, to calculate the probability of a potential operator with the consensus pentamer sequences at a particular genomic position, $P_{\text{perfect operator}}$, to occur (the identity of 10 basepairs is specified), we use

$P_{perfect\ operator} = 2\ (pAT^8\ pCG^2)$, (Equation S3)

where pAT is percent of A/T basepairs in the genome (16.7%) and pCG is percent of C/G basepairs in the genome (33.3%). The factor of 2 accounts for the operator occurring in either orientation. Using the values for the *P. aeruginosa* genome, the probability of a zero mutant operator is approximately 10^{-7}.

To estimate the number of potential zero mutant operators expected by chance in the genome, we multiply the probability of a zero mutant operator by the number of basepairs in the genome since each position in the genome is a potential starting sites for the operator sequence. Similar calculations estimate the number of 1, 2, and 3 mutation operators in the genome we should expect by chance. For these calculations we take into account that a mutation can occur in any of the 10 repeated pentamer positions. For example to calculate the probability of an operator with 1 pentamer mutation we use,

$P_{1\ mutation\ operator} = 2\ (\ 8(pAT^7\ pCG^2\ (1\text{-}pAT)\ ) + 2(pAT^8\ pCG\ (1\text{-}pCG)\ )\ )$. (Equation S4)

The factors of 8 and 2 inside the parentheses account for the number of ways in which a mutation in an A/T or a G/C could occur, respectively, and the (1-pAT) term account for the probability that the mutated base is something other than the base in the consensus sequence. The results of these calculations were verified using a simulation that generated 10,000,000 random 10 basepair sequences and calculated the probability of sequences with 10, 9, 8, or 7 identical basepairs as compared to either the consensus operator sequence or its reverse complement.

As shown in Figure S1, there are more pentamer regions without mutations than expected in the genome and fewer operators with 1, 2, or 3 pentamer mutations than would be expected by chance.

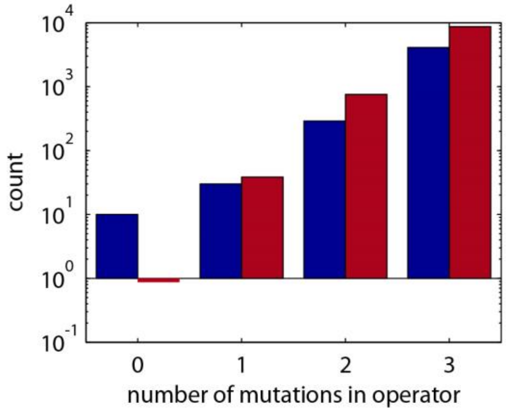

Figure S1: The distribution of potential operators containing 0, 1, 2, and 3 mutations in the ten basepairs of the repeated pentamer. Blue bars show the actual distribution in the genome of *P. aeruginosa* and the red bars show the number of expected operators that would occur by chance in a genome of the same size with a GC content of 66%.

Influence of pentamer orientation on BqsR-mediated gene regulation

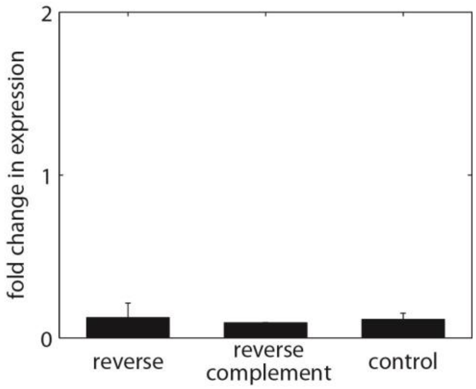

Figure S2: Changing the upstream pentamer of the operator shown in Figure 2A to the reverse (TTAAG to GAATT) or the reverse complement (TTAAG to CTTAA) reduced the fold change in expression to the level of the negative control, in which the upstream pentamer was deleted. Error bars show standard error.

Distribution of Binding Sites in the Genome

To put the distribution of overlapping and clustered BqsR binding sites shown in Figure 4 into context, calculations estimated the occurrences of overlapping and clustered binding sites. There are 432 potential BqsR operators with up to 2 pentamer mutations throughout the genome of *P. aeruginosa*. To estimate the extent of operator clustering we might expect by chance, Matlab simulations were run to calculate the average cluster sizes when 432 operators were inserted into a genome of size 6,537,647 basepairs. This genome contained genes in the same locations as the *P. aeruginosa*. The randomly inserted operators each have a unique starting position, but partial overlap of operator sequences was allowed. Each operator was assigned a random orientation. The operators could be inserted into any position within the genome. Five such random operator distributions were created and analyzed.

In these five random distributions, no overlapping binding sites were observed, that is repeats of more than two pentamers spaced by 6 basepairs with identical orientation. Therefore the repeats of up to 5 pentamers found in the actual genome, including 7 such repeats with 3 pentamers in a row, were unlikely to occur by chance.

As shown in Figure 4A, the real genome has much larger clusters of potential operators within the same regulatory region. Cluster size is the number of operators within 600 basepairs upstream of the gene coding sequence, regardless of operator orientation or relative spacing. Randomly placed operators only occasionally resulted in a cluster of only 2 operators, whereas the real genome contains several clusters of more than 4 operators in the same regulatory region.

Predicting gene expression from operator sequence

In our experiments we are measuring the fold change in gene expression upon mutating an operator sequence from its wild-type sequence. From this fold change we can calculate the relative change in the operator binding energy as a result of the change in the operator sequence.

The model is derived as previously described for the promoters such as the *lac* promoter for *E. coli* [5]. Briefly, in this model the rate of transcription is proportional to the probability of finding RNA polymerase bound to the promoter. The probability of finding the polymerase on the promoter is calculated using a thermodynamic quasi-equilibrium model. In the model the promoter has several possible microstates that are defined by the configuration of the RNA polymerase and the transcription factors on the promoter. The probability of a specific configuration is related to the copy numbers and binding energies of the proteins bound to the promoter region. These microstates and related Boltzmann terms are shown in Figure S3 for the BqsR binding promoter we are using is this study. In these Boltzmann weights, P is the number of RNA polymerase molecules per cell, A is the number of BqsR transcription factors per cell, E_a is the binding energy of BqsR to its operator sequence, E_p is the binding energy of RNA polymerase to its promoter sequence, E_c accounts for cooperativity between BqsR and RNA polymerase, N_{NS} is the number of nonspecific binding sites in the genome, k_B is the Boltzmann constant, and T is temperature.

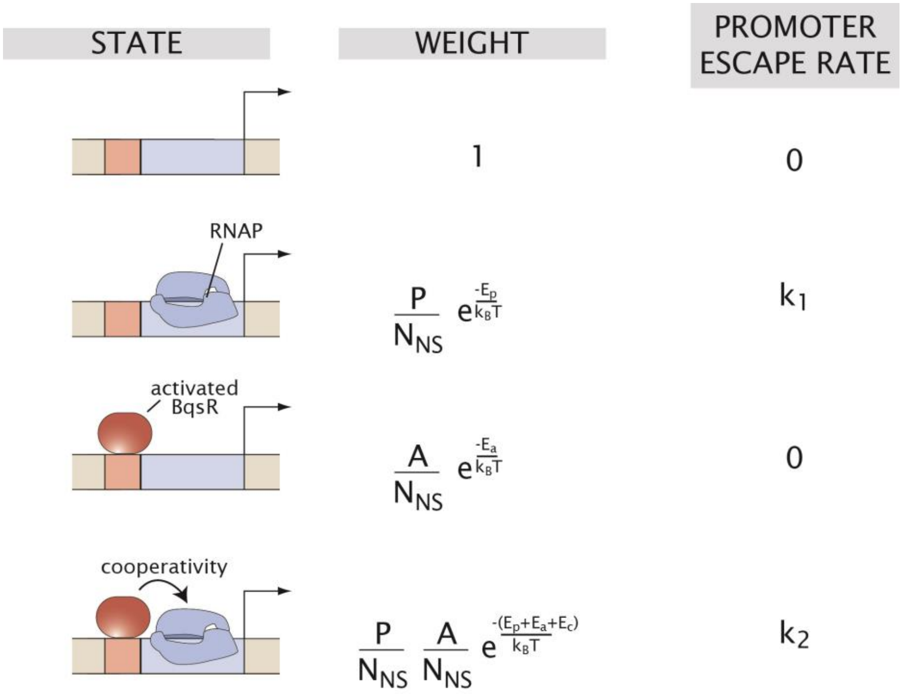

Figure S3: States and weights for BqsR-mediated gene regulation. In the bottom state there is cooperativity between RNA polymerase and BqsR, resulting in increased occupancy of the promoter by RNA polymerase in the presence of BqsR.

Another parameter which determines the production rate of mRNA is the rate constant for transcription of each promoter microstate. Any state in which the RNA polymerase is not bound has a transcriptional rate constant of zero. Each state in which the RNA polymerase is bound has a non-zero transcriptional rate constant, as listed in Figure S3.

From the Boltzmann weights listed in Figure S3, we can now derive the overall rate of transcription. The transcription rate is the sum of the probability of each state, p_i, times the rate of promoter escape, r_i. This gives,

$$transcription\ rate = \sum p_i r_i. \qquad \text{(Equation S5)}$$

The probability of a state is the Boltzmann weight for that state divided by the sum of the Boltzmann weights for every possible microstate,

$$p_i = \frac{w_i}{\sum w_i}. \qquad \text{(Equation S6)}$$

From these definitions we can derive the transcription rate for our particular system as,

$$transcription\ rate = \frac{k_1 \frac{P}{N_{NS}} e^{\frac{-E_p}{k_B T}} + k_2 \frac{P}{N_{NS}} \frac{A}{N_{NS}} e^{\frac{-(E_p+E_a+E_c)}{k_B T}}}{1 + \frac{P}{N_{NS}} e^{\frac{-E_p}{k_B T}} + \frac{P}{N_{NS}} \frac{A}{N_{NS}} e^{\frac{-(E_p+E_a+E_c)}{k_B T}} + \frac{A}{N_{NS}} e^{\frac{-E_a}{k_B T}}}. \qquad \text{(Equation S7)}$$

Now consider we make a change to the operator sequence to which the transcription factor BqsR binds. We assume that this change in the operator sequence only influences the binding energy of BqsR to its operator and has no influence on both the transcription rate constant and the cooperativity term from the RNA polymerase and BqsR bound microstate. The binding energy of BqsR to the mutated operator sequence we call E_m. From this definition we can calculate the fold change in the transcription rate upon mutating the BqsR operator as,

$$fold\ change = \frac{\dfrac{k_1 \frac{P}{N_{NS}} e^{\frac{-E_p}{k_B T}} + k_2 \frac{P}{N_{NS}} \frac{A}{N_{NS}} e^{\frac{-(E_p+E_a+E_c)}{k_B T}}}{1+\frac{P}{N_{NS}} e^{\frac{-E_p}{k_B T}} + \frac{P}{N_{NS}} \frac{A}{N_{NS}} e^{\frac{-(E_p+E_a+E_c)}{k_B T}} + \frac{A}{N_{NS}} e^{\frac{-E_a}{k_B T}}}}{\dfrac{k_1 \frac{P}{N_{NS}} e^{\frac{-E_p}{k_B T}} + k_2 \frac{P}{N_{NS}} \frac{A}{N_{NS}} e^{\frac{-(E_p+E_m+E_c)}{k_B T}}}{1+\frac{P}{N_{NS}} e^{\frac{-E_p}{k_B T}} + \frac{P}{N_{NS}} \frac{A}{N_{NS}} e^{\frac{-(E_p+E_m+E_c)}{k_B T}} + \frac{A}{N_{NS}} e^{\frac{-E_m}{k_B T}}}}. \qquad \text{(Equation S8)}$$

Experimentally, this fold change is equivalent to the ratio of gene expression for cells containing the wild-type operator sequence to cells containing the mutated operator. This equation can be simplified to,

$$fold\ change = \frac{\dfrac{\frac{P}{N_{NS}} e^{\frac{-E_p}{k_B T}} (1+\frac{k_2}{k_1} \frac{A}{N_{NS}} e^{\frac{-(E_a+E_c)}{k_B T}})}{(1+\frac{P}{N_{NS}} e^{\frac{-E_p}{k_B T}})(1+\frac{A}{N_{NS}} e^{\frac{-(E_a+E_c)}{k_B T}})}}{\dfrac{\frac{P}{N_{NS}} e^{\frac{-E_p}{k_B T}} (1+\frac{k_2}{k_1} \frac{A}{N_{NS}} e^{\frac{-(E_m+E_c)}{k_B T}})}{(1+\frac{P}{N_{NS}} e^{\frac{-E_p}{k_B T}})(1+\frac{A}{N_{NS}} e^{\frac{-(E_m+E_c)}{k_B T}})}} = \frac{\dfrac{(1+\frac{k_2}{k_1} \frac{A}{N_{NS}} e^{\frac{-(E_a+E_c)}{k_B T}})}{(1+\frac{A}{N_{NS}} e^{\frac{-(E_a+E_c)}{k_B T}})}}{\dfrac{(1+\frac{k_2}{k_1} \frac{A}{N_{NS}} e^{\frac{-(E_m+E_c)}{k_B T}})}{(1+\frac{A}{N_{NS}} e^{\frac{-(E_m+E_c)}{k_B T}})}}. \qquad \text{(Equation S9)}$$

Making the approximation that the transcription rate in the BqsR bound case is much larger than the transcription rate when only the RNA polymerase is bound, i.e. $\frac{k_2}{k_1} \frac{A}{N_{NS}} e^{\frac{-(E_a+E_c)}{k_B T}} \gg 1$ and $\frac{k_2}{k_1} \frac{A}{N_{NS}} e^{\frac{-(E_m+E_c)}{k_B T}} \gg 1$, we can further simplify the fold change expression,

$$fold\ change = \frac{\dfrac{\frac{A}{N_{NS}} e^{\frac{-E_a}{k_B T}})}{(1+\frac{A}{N_{NS}} e^{\frac{-E_a}{k_B T}})}}{\dfrac{\frac{A}{N_{NS}} e^{\frac{-E_m}{k_B T}})}{(1+\frac{A}{N_{NS}} e^{\frac{-E_m}{k_B T}})}}. \qquad \text{(Equation S10)}$$

This approximation is well supported by the experimental data in Figure S4 which shows the expression of the reporter gene LacZ is low the in the absence of strong BqsR activation.

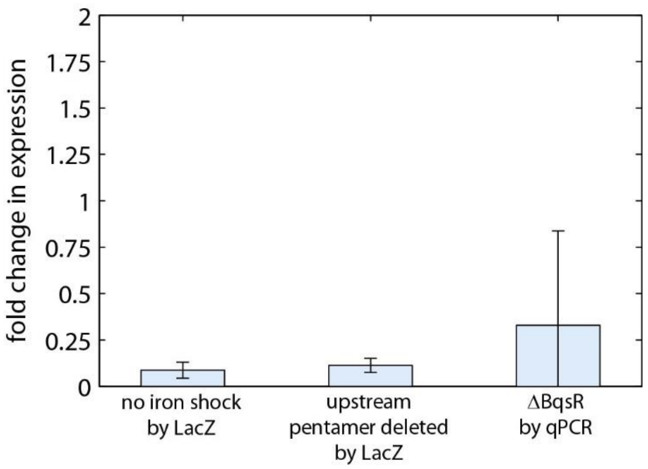

Figure S4: Fold change in expression in the absence of strong BqsR activity. Without iron shock, no extra iron is added during the shock, the fold change in expression is 9 +- 4%. Upon mutating all five bases of the upstream operator, the fold change in expression fell to 11 +- 4%. Given the mutations made in the pentamer (TTAAG to ACTCA), using the energy matrix in Figure 5B we would calculate residual expression of 7%. These measurements were taken using the LacZ gene reporter for the mutated PA14_04180 promoter region containing only two pentamers. The final bar shows the change in gene expression for the wild-type version of gene PA14_04180 upon deleting BqsR from the genome, measured using qPCR. Together these indicate the gene expression level of PA14_04180 in the absence of BqsR-mediated activation should be negligible.

With this further simplification the transcription rate constants have now been removed from the fold change expression. The remaining expression has two interesting limits. In the limit that $\frac{A}{N_{NS}} e^{\frac{-E_a}{k_B T}} \gg 1$ and $\frac{A}{N_{NS}} e^{\frac{-E_m}{k_B T}} \gg 1$, the fold change goes to 1. In this limit, the probability of BqsR being bound is very large, therefore it is not surprising that a change in the BqsR binding energy may not influence the fold change. We could call this the operator saturation limit. The more interesting limit is when $\frac{A}{N_{NS}} e^{\frac{-E_a}{k_B T}} \ll 1$ and $\frac{A}{N_{NS}} e^{\frac{-E_m}{k_B T}} \ll 1$, when we would not expect to always find BqsR bound to the operator. In other words, we still expect some "action" from changes in BqsR copy number and binding energy. In the limit of not saturating the operator the fold change simplifies to,

$$fold\ change = e^{\frac{-(E_a - E_m)}{k_B T}} \qquad \text{(Equation S11)}$$

or

$$-\ln(fold\ change) = \frac{\Delta E_{mut}}{k_B T}, \qquad \text{(Equation S12)}$$

where ΔE_{mut} is the change in the binding energy due to the mutation. From the fold change measurement we can calculate the change in the BqsR binding energy caused by a mutation in the operator.

In the reverse case, we can also then back calculate the fold change for a given operator if we know how each basepair mutation influences the binding energy. This assumes that each individual mutation in the operator sequence has an additive effect on the operator binding energy, a first approximation that has proven useful in previous examples [6, 7].

To be more explicit, starting from the wild-type operator, if we have 3 point mutations in the operator we would expect the fold change to be,

$$fold\ change = e^{\frac{-(\Delta E_{m1}+\Delta E_{m2}+\Delta E_{m3})}{k_B T}} = e^{\frac{-\Delta E_{m1}}{k_B T}} e^{\frac{-\Delta E_{m2}}{k_B T}} e^{\frac{-\Delta E_{m3}}{k_B T}}. \quad \text{(Equation S13)}$$

In Equation S13 ΔE_{mi} is the change in the binding energy due to the ith mutation in the operator sequence. Of course we should keep in mind that this fold change is relative to the gene expression from the "wild-type" sequence and not an exact level of expression (unless of course we knew the precise level of gene expression in the wild-type case).

The fold changes due to each change in the operator sequence are multiplied together. For example if mutation 1 has a fold change of 0.8 and mutation 2 has a fold change of 0.5, the predicted fold change for an operator with both mutations would be the product of the two fold changes or 0.4.

Deriving the energy matrix for the spacer region

To derive the "best-fit" matrix, first we assume the sequence of the spacer region does not influence expression. This is equivalent to saying all the bases of the spacer region contribute 0 $k_B T$ to the binding energy. From this starting matrix, we predict the expression level for synthetic promoters containing the 7 spacer sequences (Fig. 3D), and calculate the deviations of the predictions from the experimental measurements. The value of the matrix at a random position is then increased or decreased by 4%, and new deviations from the measured expression levels are calculated. If the prediction was more accurate, the change to the energy matrix was retained. If the prediction was less accurate, there was a 1% chance the change was kept. This process was iterated 10,000 times to generate a final matrix that is the best fit to the experimental data. To ensure that the fitting procedure did not get stuck at a local minimum, the whole process was then repeated from the all zeros spacer energy matrix 100 times and these 100 final matrices were averaged together. Figure S5 shows the final energy matrix reached an optimum in less than 10,000 iterations.

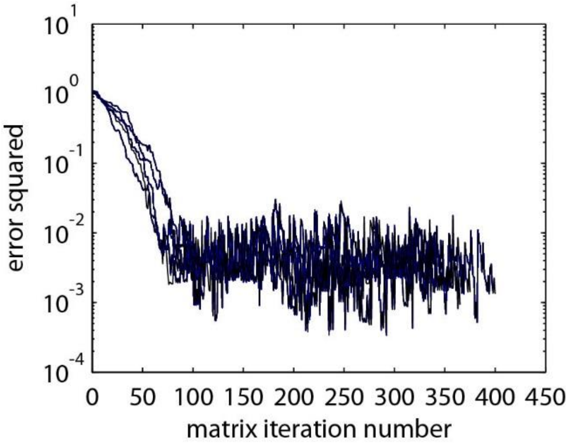

Figure S5: In fitting the energy matrix for the middle 6 basepairs of the operator, the value of the binding energy matrix for a random basepair was increased or decreased by 4% and the resultant error in the prediction using this new energy matrix was calculated. As mutations accumulated, the square in the error quickly reduced to a level less than 10^{-3}. The graph above shows the squared error as a function of the number of changes for 5 independent fits. The spacer region for the energy matrix reported in Figure 5B averages together 100 such replicate fits.

Distribution of operator strengths and gene expression

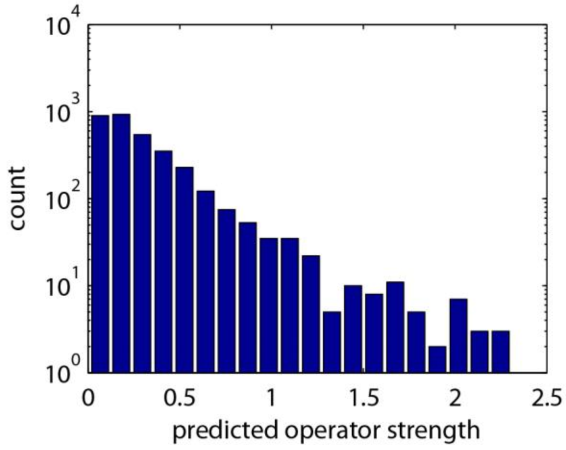

Figure S6: The distribution of predicted operator affinities for all 3347 potential operators containing less than 3 pentamer region mutations found within 600 basepairs upstream of the coding sequence. Binding energies for each operator were calculated using the energy matrix in Figure 5B of the main text. The starting sequence shown in Figure 5A has a predicted operator strength of 1. The model assumes operator strength is proportional fold change in expression.

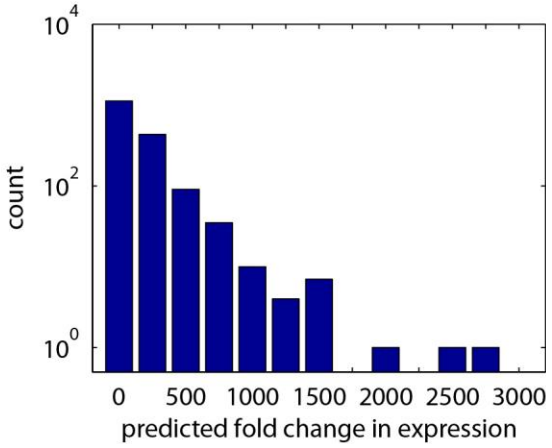

Figure S7: The distribution of predicted fold change in gene expression for all genes containing a potential BqsR operator with fewer than 3 pentamer mutations within 600 basepairs upstream of the coding sequence. Predictions are made using the energy matrix from Fig. 5B and scaled such that the prediction for the reference gene PA14_04180 matches the experimentally measured value.

Additive approximation for multi-operator promoters

To predict expression from promoter regions containing multiple BqsR binding sites, we used an additive model. From the results of Figure 4, we found that having multiple binding sites in the same promoter region leads to increased expression in the case of the PA14_04180 promoter. We approximated the combined influence of multiple operators at the same promoter using an additive model in which,

$$fold\ change\ in\ expression_{total} = \sum fold\ change_i. \qquad \text{(Equation S14)}$$

Fold change$_i$ is the fold change due to operator *i*, where fold change is calculated using Equation 2. This additive approximation is equivalent to assuming the two operators act independently on gene expression.

To access the consequences of this approximation, we next calculated the total fold change in gene expression for a promoter containing two operators when using the additive approximation versus a thermodynamic model which incorporates the possibility of having both operators occupied simultaneously.

For the additive case, the influence of each operator on transcription can be calculated using,

$$transcription\ rate = \frac{k_1 \frac{P}{N_{NS}} e^{\frac{-E_p}{k_B T}} + k_2 \frac{P}{N_{NS}} \frac{A}{N_{NS}} e^{\frac{-(E_p+E_a+E_c)}{k_B T}}}{1+\frac{P}{N_{NS}} e^{\frac{-E_p}{k_B T}} + \frac{P}{N_{NS}} \frac{A}{N_{NS}} e^{\frac{-(E_p+E_a+E_c)}{k_B T}} + \frac{A}{N_{NS}} e^{\frac{-E_a}{k_B T}}}. \text{ (Equation S15)}$$

where P is the number of RNA polymerase molecules per cell, A is the number of BqsR transcription factors per cell, E_a is the binding energy of BqsR to its operator sequence, E_p is the binding energy of RNA polymerase to its promoter sequence, E_c accounts for cooperativity between BqsR and RNA polymerase, k_i is the rate of transcription from state i, N_{NS} is the number of nonspecific binding sites in the genome, k_B is the Boltzmann constant, and T is temperature.

When not using the additive approximation, we derive the transcription rate from the states and weights shown in Figure S8. As can be seen in these states, the two operators have different binding energies, E_{a1} and E_{a2}. The energy that accounts for cooperativity between BqsR and RNA polymerase is not dependent on whether operator 1, operator 2, or both operators are bound.

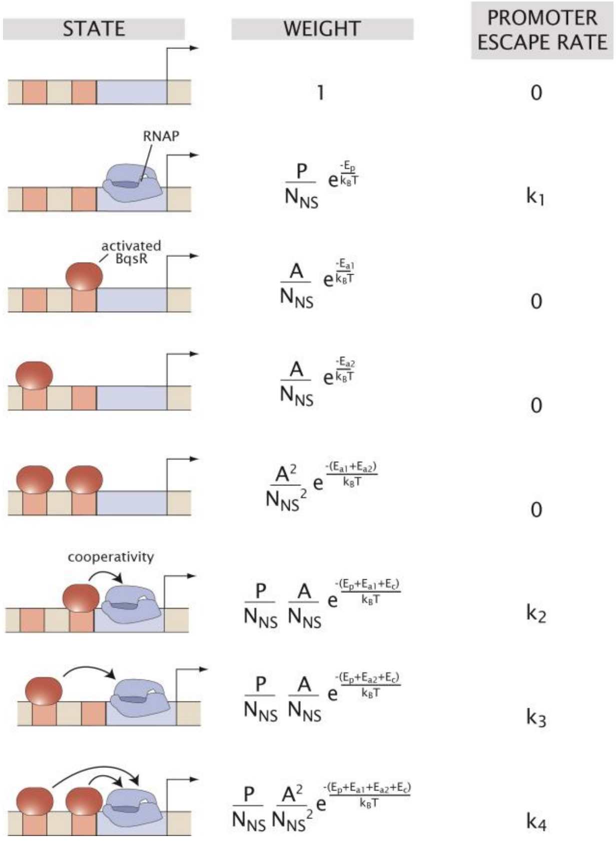

Figure S8: The states and weights for a promoter containing two BqsR operators.

From the weights in Figure S8, we derive the transcription rate for the case of two BqsR operators to be,

$transcription\ rate =$

$$\frac{k_1 \frac{P}{N_{NS}} e^{\frac{-E_p}{k_B T}} + k_2 \frac{PA}{N_{NS}^2} e^{\frac{-(E_p+E_{a1}+E_c)}{k_B T}} + k_3 \frac{PA}{N_{NS}^2} e^{\frac{-(E_p+E_{a2}+E_c)}{k_B T}} + k_4 \frac{PA^2}{N_{NS}^3} e^{\frac{-(E_p+E_{a1}+E_{a2}+E_c)}{k_B T}}}{1+\frac{P}{N_{NS}} e^{\frac{-E_p}{k_B T}} + \frac{A}{N_{NS}} e^{\frac{-E_{a1}}{k_B T}} + \frac{A}{N_{NS}} e^{\frac{-E_{a2}}{k_B T}} + \frac{A^2}{N_{NS}} e^{\frac{-(E_{a1}+E_{a2})}{k_B T}} + \frac{PA}{N_{NS}^2} e^{\frac{-(E_p+E_{a1}+E_c)}{k_B T}} + \frac{PA}{N_{NS}^2} e^{\frac{-(E_p+E_{a2}+E_c)}{k_B T}} + \frac{PA^2}{N_{NS}^3} e^{\frac{-(E_p+E_{a1}+E_{a2}+E_c)}{k_B T}}}.$$

(Equation S16)

We calculated the ratios of transcription rates for the additive model, using Equations S14 and S15, over the full thermodynamic model, using Equation S16. The results are shown in Figure S9.

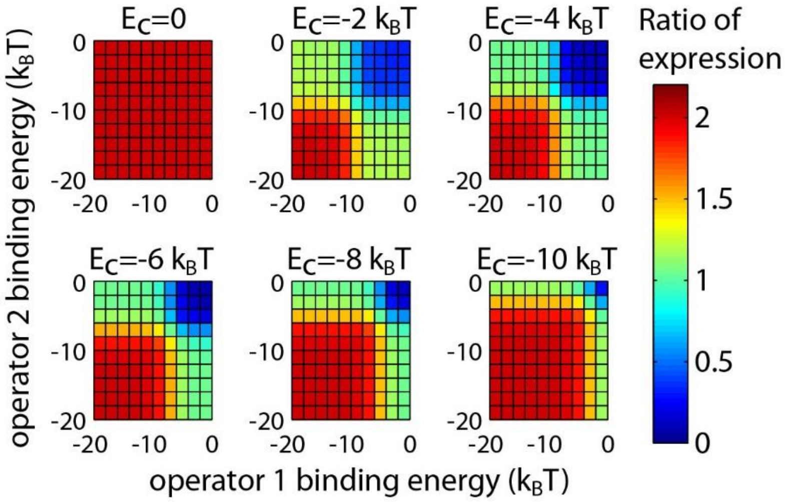

Figure S9: The ratio of expression levels for a model which assumes operators act independently, the additive model, to a model which directly incorporates the states in which both operators are bound. Calculations are for a promoter containing two operators. The axes of each plot are the binding energies of the two operators. For these calculations the number of BqsR per cell was 100, the number of nonspecific binding sites was 5×10^6, the RNA polymerase binding energy was -3 $k_B T$, and the number of RNA polymerase per cell was 10,000. In these calculations we assumed the promoter escape rates, *k_i's*, for all states were equal.

In Figure S9, the interaction energies range from 0 to -10 $k_B T$, and the operator binding energies range from 0 to -20 $k_B T$. We found that when both operator binding energies are strong, <-10 $k_B T$, the prediction using the additive model will be within a factor of two of the prediction using the full thermodynamic model whose states are listed in Figure S8. For intermediate interaction energies, -2 $k_B T$ to -8 $k_B T$, the additive model is close to the full thermodynamic model when one of the operators had a strong binding energy and the other had a weak binding energy. The calculations with the additive model were particularly poor when both operators had very weak binding, approximately more than -5 $k_B T$. Given that typical binding energy are -5 to -15 $k_B T$, and interaction energies with RNA polymerases have been observed to be 0 to -10 $k_B T$, we expect calculations made for a promoter containing two operators using the additive model to be within a factor of 2 of calculations using a full thermodynamic model. Therefore the additive model is unlikely to contribute significantly to the general overestimate of expression levels observed in Figure 6 of the main text.

Single operator predictions

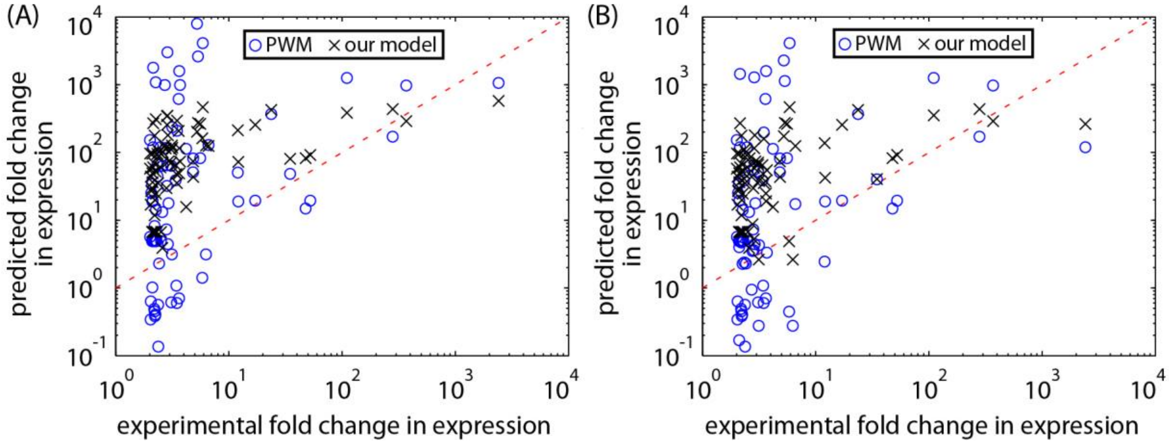

Figure S10: Comparison of predictions to experimental measurements of gene expression. As in Figure 6 of the main text, the predictions use the BqsR binding matrices found in Figure 5. Unlike in Figure 6, in (A) we only use the strongest binding potential operator and in (B) we only use the closest potential operator to predict expression. All operators used contain up to 3 mutations and are found between -600 and 0 bases of the coding sequence.

Gene expression predictions taking into account operator orientation

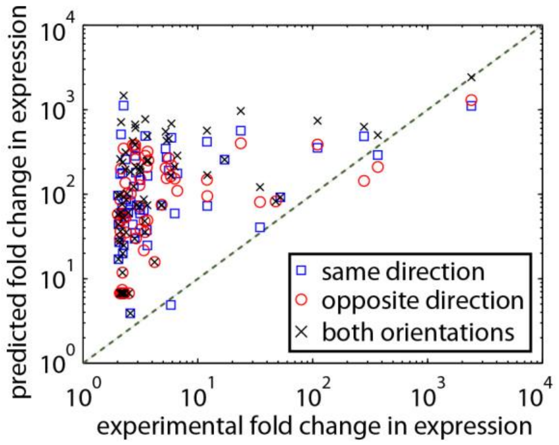

Figure S11: Predictions of gene expression for the same set of genes shown in Figure 6 when taking operator direction into account. The black x's replot the original predictions from Figure 6B which allow the potential operators to be in either orientation relative to the direction of the regulated gene. The blue squares show the predictions for the same genes using only those operators that have the same orientation of the regulated gene, and the red circles show the predictions using only those operators that have the opposite orientation of the regulated gene. For some of the predicted genes, all of the operator face in the same direction, therefore the red circles and blue squares each contain a subset of the predictions made using both orientations.

Measuring gene expression in response to ferrous iron shock

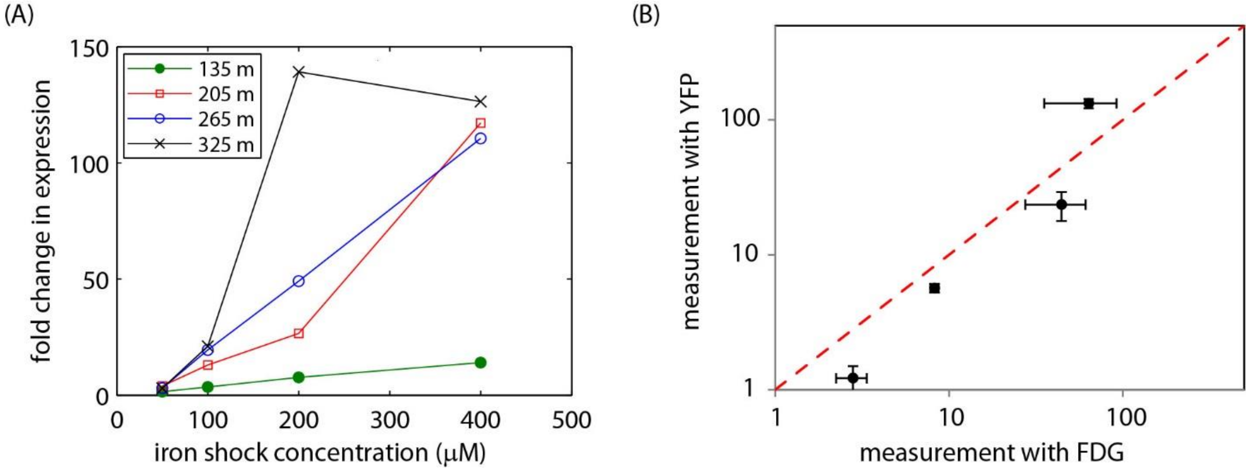

Figure S12: (A) Cells containing a *lacZ* reporter attached to the promoter region of gene PA_04180 were shocked with ferrous iron concentrations between 50 and 400 μM for either 135, 205, 265, or 325 minutes (see legend). Following the shock, expression levels of *lacZ* were quantified using the fluorogenic indicator FDG. It was found that upon shocking with 400 μM ferrous iron, the fold change reached a maximum in less than 205 minutes. (B) Comparison of the gene expression measured using the FDG assay and the LacZ reporter to a YFP reporter. Measurements were performed on aerobically grown *E. coli* strains containing a single *lac* operator at +11 relative to the transcription start site and the wildtype number of Lac repressors per cells (see [8] for details). The four data points correspond to construct containing the O1, O2, O3, and Oid *lac* operator.

Comparison to other operons

We compare the genes experimentally found to be under BqsR control with 13 experimentally validated *P. aeruginosa* regulons from the literature. Most of these regulons did not significantly overlap with the BqsR regulon, see Table S1. Figure 7C shows the results of a few of these comparisons. For some transcription factors, the regulon was reported more than once. Two published datasets for Fur were compared to the BqsR regulon. Only one dataset significantly overlapped with the BqsR regulon (Palma et al. 2003) and the expression of all of the overlapping TUs were only marginally perturbed by Fur. Two methods for finding the PqsR regulon were used (Déziel et al. 2005; Bredenbruch et al. 2006) and both regulons overlapped with TUs we predicted to be BqsR regulated but did not appear in the RNAseq data with statistical significance. Anr represses all of the genes (Trunk et al. 2010) that statistically significantly overlap with predicted BqsR TUs, but did not appear in the RNAseq data. RpoN activates many of the genes (Damron et al. 2012) that we observed in the experimental data but were not predicted using our binding energy matrix, called the false negatives in the main text.

Table S1. Known *P. aeruginosa* regulons comparison to predicted BqsR regulon. *p-value < 0.05

regulon	total transcription units in regulon	overlap with false positive predictions	significance of overlap	overlap with false negative predictions	significance of overlap	Reference
Anr	47	12	*4.01E-12	0		9
Dnr	7	0		0		9
ArgR	22	1	7.56E-02	0		10
Fur 1	84	0		0		11
Fur 2	137	7	*7.06E-03	0		12
MexT	101	1	6.28E-01	0		13
LasR	55	1	3.18E-01	0		14
PqsR 1	78	8	*2.65E-05	0		15
PqsR 2	107	9	*5.90E-05	0		16
All quorum sensing	77	2	2.15E-01	0		14
RhlR	30	1	1.28E-01	0		14
RpoS exponential	49	1	2.72E-01	0		14
RpoS stationary	21	0		0		14
RsmA	375	10	1.52E-01	1	4.10E-01	17
Vfr	139	1	7.93E-01	1	9.10E-02	18
RpoN all	659	18	7.97E-02	5	*2.29E-02	19
RpoN induced	335	10	8.47E-02	3	*2.93E-02	19
RpoN repressed	327	8	2.38E-01	2	1.13E-01	19

Supplemental Material References:

1. Hertz, G.Z. & Stormo, G.D. Identifying DNA and protein patterns with statistically significant alignments of multiple sequences. *Bioinformatics* **15**, 563-577 (1999).
2. Vilar, J.M.G. Accurate Prediction of Gene Expression by Integration of DNA Sequence Statistics with Detailed Modeling of Transcription Regulation. *Biophysical Journal* **99**, 2408-2413 (2010).
3. Winsor, G.L. et al. Pseudomonas Genome Database: improved comparative analysis and population genomics capability for Pseudomonas genomes. *Nucleic Acids Research* **39**, D596-D600 (2011).
4. Grant, C.E., Bailey, T.L. & Noble, W.S. FIMO: scanning for occurrences of a given motif. *Bioinformatics* **27**, 1017-1018 (2011).
5. Razo-Mejia, M. et al. Comparison of the theoretical and real-world evolutionary potential of a genetic circuit. *Physical Biology* **11** (2014).

6. Brewster, R.C., Jones, D.L. & Phillips, R. Tuning Promoter Strength through RNA Polymerase Binding Site Design in Escherichia coli. *Plos Computational Biology* **8**, e1002811-e1002811 (2012).
7. Kinney, J.B., Murugan, A., Callan, C.G., Jr. & Cox, E.C. Using deep sequencing to characterize the biophysical mechanism of a transcriptional regulatory sequence. *Proceedings of the National Academy of Sciences of the United States of America* **107**, 9158-9163 (2010).
8. Garcia, H.G. & Phillips, R. Quantitative dissection of the simple repression input-output function. *Proceedings of the National Academy of Sciences of the United States of America* **108**, 12173-12178 (2011).
9. Trunk, K. et al. Anaerobic adaptation in Pseudomonas aeruginosa: definition of the Anr and Dnr regulons. *Environmental Microbiology* **12**, 1719-1733 (2010).
10. Lu, C.-D., Yang, Z. & Li, W. Transcriptome Analysis of the ArgR Regulon in Pseudomonas aeruginosa. *Journal of Bacteriology* **186**, 3855-3861 (2004).
11. Ochsner, U.A., Wilderman, P.J., Vasil, A.I. & Vasil, M.L. GeneChip® expression analysis of the iron starvation response in Pseudomonas aeruginosa: identification of novel pyoverdine biosynthesis genes. *Molecular Microbiology* **45**, 1277-1287 (2002).
12. Palma, M., Worgall, S. & Quadri, L.N. Transcriptome analysis of the Pseudomonas aeruginosa response to iron. *Archives of Microbiology* **180**, 374-379 (2003).
13. Tian, Z.-X. et al. Transcriptome profiling defines a novel regulon modulated by the LysR-type transcriptional regulator MexT in Pseudomonas aeruginosa. *Nucleic Acids Research* **37**, 7546-7559 (2009).
14. Schuster, M. & Greenberg, E. Early activation of quorum sensing in Pseudomonas aeruginosa reveals the architecture of a complex regulon. *BMC Genomics* **8**, 1-11 (2007).
15. Bredenbruch, F., Geffers, R., Nimtz, M., Buer, J. & Häussler, S. The Pseudomonas aeruginosa quinolone signal (PQS) has an iron-chelating activity. *Environmental Microbiology* **8**, 1318-1329 (2006).
16. Déziel, E. et al. The contribution of MvfR to Pseudomonas aeruginosa pathogenesis and quorum sensing circuitry regulation: multiple quorum sensing-regulated genes are modulated without affecting lasRI, rhlRI or the production of N-acyl- l-homoserine lactones. *Molecular Microbiology* **55**, 998-1014 (2005).
17. Burrowes, E., Baysse, C., Adams, C. & O'Gara, F. Influence of the regulatory protein RsmA on cellular functions in Pseudomonas aeruginosa PAO1, as revealed by transcriptome analysis. *Microbiology* **152**, 405-418 (2006).
18. Wolfgang, M.C., Lee, V.T., Gilmore, M.E. & Lory, S. Coordinate Regulation of Bacterial Virulence Genes by a Novel Adenylate Cyclase-Dependent Signaling Pathway. *Developmental Cell* **4**, 253-263.
19. Damron, F.H. et al. Analysis of the Pseudomonas aeruginosa Regulon Controlled by the Sensor Kinase KinB and Sigma Factor RpoN. *Journal of Bacteriology* **194**, 1317-1330 (2012).
